# Familial misophonia or selective sound sensitivity syndrome : evidence for autosomal dominant inheritance?^☆^

**DOI:** 10.1016/j.bjorl.2017.06.014

**Published:** 2017-07-29

**Authors:** Tanit Ganz Sanchez, Fúlvia Eduarda da Silva

**Affiliations:** a Instituto Ganz Sanchez, São Paulo, SP, Brazil; b Universidade de São Paulo (USP), Faculdade de Medicina, Departamento de Otorrinolaringologia, São Paulo, SP, Brazil; c Universidade de São Paulo (USP), Pós-graduação em Ciências da Reabilitação, São Paulo, SP, Brazil

**Keywords:** Misophonia, Tinnitus, Hyperacusis, Heredity, Autosomal dominant inheritance, Misofonia, Zumbido, Hiperacusia, Hereditariedade, Herança autossômica dominante

## Abstract

**Introduction:**

Misophonia is a recently described, poorly understood and neglected condition. It is characterized by strong negative reactions of hatred, anger or fear when subjects have to face some selective and low level repetitive sounds. The most common ones that trigger such aversive reactions are those elicited by the mouth (chewing gum or food, popping lips) or the nose (breathing, sniffing, and blowing) or by the fingers (typing, kneading paper, clicking pen, drumming on the table). Previous articles have cited that such individuals usually know at least one close relative with similar symptoms, suggesting a possible hereditary component.

**Objective:**

We found and described a family with 15 members having misophonia, detailing their common characteristics and the pattern of sounds that trigger such strong discomfort.

**Methods:**

All 15 members agreed to give us their epidemiological data, and 12 agreed to answer a specific questionnaire which investigated the symptoms, specific trigger sounds, main feelings evoked and attitudes adopted by each participant.

**Results:**

The 15 members belong to three generations of the family. Their age ranged from 9 to 73 years (mean 38.3 years; median 41 years) and 10 were females. Analysis of the 12 questionnaires showed that 10 subjects (83.3%) developed the first symptoms during childhood or adolescence. The mean annoyance score on the Visual Analog Scale from 0 to 10 was 7.3 (median 7.5). Individuals reported hatred/anger, irritability and anxiety in response to sounds, and faced the situation asking to stop the sound, leaving/avoiding the place and even fighting. The self-reported associated symptoms were anxiety (91.3%), tinnitus (50%), obsessive-compulsive disorder (41.6%), depression (33.3%), and hypersensitivity to sounds (25%).

**Conclusion:**

The high incidence of misophonia in this particular familial distribution suggests that it might be more common than expected and raises the possibility of having a hereditary etiology.

## Resumo

**Introdução:**

A misofonia é uma condição recentemente descrita, mal compreendida e negligenciada. É caracterizada por fortes reações negativas de ódio, raiva ou medo quando os indivíduos precisam enfrentar alguns sons repetitivos seletivos e de baixa intensidade. Os mais comuns que desencadeiam tais reações aversivas são aqueles provocados pela boca (mascar goma ou mastigar comida, estalar os lábios) ou nariz (respirando, cheirando e soprando) ou pelos dedos (digitando, amassando papel, clicando a caneta, tamborilando na mesa). Artigos anteriores citam que esses indivíduos geralmente conhecem pelo menos um parente próximo com sintomas semelhantes, sugerindo um possível componente hereditário.

**Objetivo:**

Encontramos e descrevemos uma família com 15 membros com misofonia, detalhando suas características comuns e o padrão de sons que desencadeiam um desconforto tão forte.

**Método:**

Todos os 15 membros concordaram em nos fornecer seus dados epidemiológicos e 12 concordaram em responder a um questionário específico que investigou os sintomas, sons de gatilho específicos, principais sentimentos evocados e atitudes adotadas por cada participante.

**Resultados:**

Os 15 membros pertencem a três gerações da família. A idade variou de 9 a 73 anos (média de 38,3 anos, mediana de 41 anos) e 10 eram mulheres. A análise dos 12 questionários mostrou que 10 indivíduos (83,3%) desenvolveram os primeiros sintomas durante a infância ou a adolescência. A média do escore de irritação na Escala Visual Analógica de 0 a 10 foi de 7,3 (mediana 7,5). Os indivíduos relataram sentimentos de ódio/raiva, irritabilidade e ansiedade em resposta a sons, e enfrentaram a situação pedindo para interromper o som, deixando/evitando o lugar e até mesmo discutindo. Os sintomas associados auto-relatados foram ansiedade (91,3%), zumbido (50%), transtorno obsessivo-compulsivo (41,6%), depressão (33,3%) e hipersensibilidade aos sons (25%).

**Conclusão:**

A alta incidência de misofonia nessa distribuição familiar em particular sugere que possa ser mais comum do que o esperado e suscita a possibilidade de haver uma etiologia hereditária.

## Introduction

Misophonia (miso = dislike; phone = sounds) is unknown among most professionals who study hearing. Also known as Selective Sound Sensitivity Syndrome (4S), it applies to patients who have aversion to very specific sounds, such as chewing, breathing, click pen, snapping lips, wheezing etc.^1^, ^2^, ^3^, ^4^, ^5^, ^6^ These are usually low level, but repetitive sounds, causing the individuals a strong, sudden, uncontrolled and disproportionate emotional reaction.

The causes and prevalence of misophonia remain unknown.^3^ However, there are online groups with thousands of members in English, Spanish and Portuguese, suggesting that it may be bigger than established by research.

Misophonia sufferers are fully aware of their abnormal reactions to sounds.^3^ They avoid situations where such particular sounds can be produced and consequently have the familial, social and professional interactions severely limited.^3^ Some subjects even feel themselves as “ridiculous”, but they cannot overcome the problem by themselves. Patients often recognize that present symptoms started during childhood/adolescence.^7^

Misophonia has some similarities with tinnitus,^3^ which is an internal sound that 10–22% individuals perceive in the ears or head.^8^, ^9^, ^10^, ^11^, ^12^ Tinnitus has been a growing phenomenon worldwide, also reaching high prevalence among children and adolescents,^13^, ^14^ which is the age range that misophonia is reported to start. It is accepted that, if tinnitus is associated with a negative connotation, the connections between auditory, limbic and autonomic systems increase^15^ and cause further nuisance, with consequent failure of the spontaneous habituation to sounds.^16^ This mechanism can also occur with the external sounds that characterize misophonia, suggesting that both conditions can evoke strong reactions to their sound triggers, either internal (tinnitus) or external (misophonia).

Some patients report at least one close relative with similar symptoms of misophonia, suggesting a possible hereditary component.^3^ The aim of this study is to describe a family with 15 members affected by misophonia, their behavioral characteristics and the pattern of sounds that evoke such unusual and strong discomfort.

## Methods

**Figure 1:**
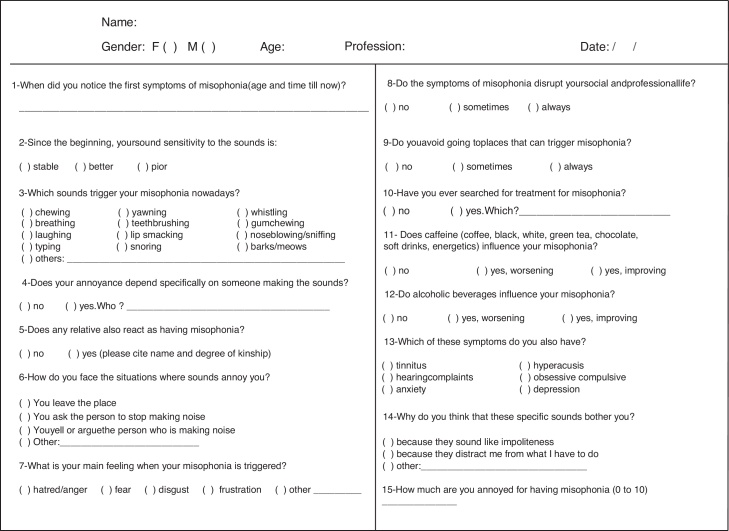
Specific questionnaire created for this research on Misophonia (Selective Sound Sensitivity Syndrome).

During the routine medical consultation of a patient with misophonia, she reported that at least seven other family members had similar symptoms. She and her family were invited to participate in a research to describe their cases. Upon signing the written consent approved by the Ethical Committee (1458/15), the survey was conducted through a questionnaire (Fig. 1), and the interviews were taken by phone, email or skype due to the long distance of their cities.

Such individuals indicated other family members with similar symptoms, who were also contacted and invited to participate under the same conditions. The family was eventually characterized as presenting 15 members affected by misophonia, who were distributed in three generations in the family tree. Among them, 12 agreed to answer the whole questionnaire.

We performed descriptive statistical analysis in different samples, according to the specific focus: the epidemiological data and the family tree included all the 15 subjects, and the data about the questionnaire included the 12 subjects.

## Results

### Epidemiological data

**Figure 2:**
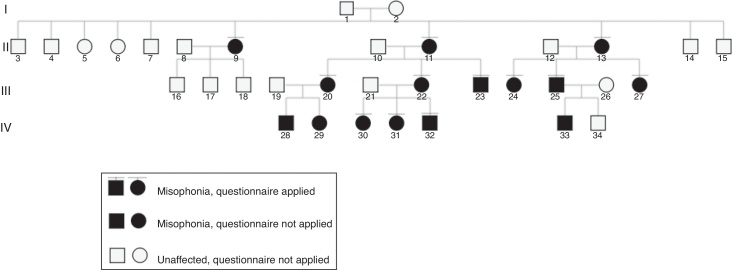
Genealogy of the family with 15 affected members with misophonia.

Fig. 2 shows the genealogy of the family members. The age ranged from 9 to 73 years (mean 38.3 and median of 41 years), 10 (66.6%) were women and 100% were Caucasians.

They live in 3 different Brazilian cities: Natal (RN), Fortaleza (CE) and São Paulo (SP). Regarding the education level, six are students, one is trading, and eight had complete superior education (two administrators, two lawyers, one engineer, one psychologist, one businesswoman and one university professor).

### Questionnaire data: onset of symptoms and evolution

**Figure 3:**
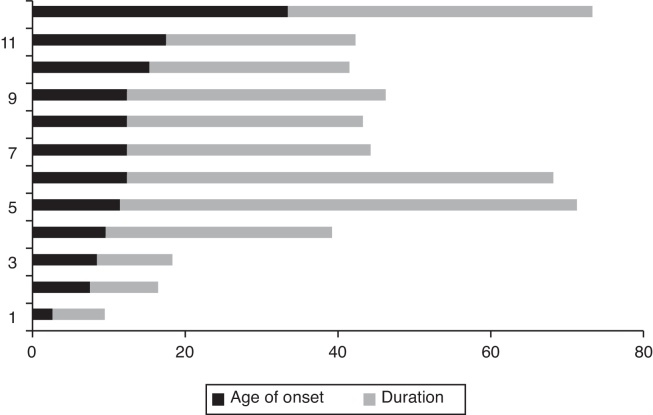
Reported age at the onset of the misophonia symptoms and time of duration till present (*n* = 12). Data is presented in descending order, considering the age of onset, and not the number in the genealogy shown in Fig. 2.

The first symptoms of misophonia started at the age 2–33 years. By adding such information to the current age of each participant, the duration of misophonia corresponded to the interval from 7 to 60 years (mean = 30 years; median = 30.5 years) (Fig. 3).

None of them had previously searched for treatment. So, the natural evolution over time showed that 7 (58.3%) subjects feel that they are worsening, 3 (25%) are stable and 2 (16.7%) had spontaneous improvement.

### Selectivity of trigger sounds, feelings and attitudes

**Figure 4:**
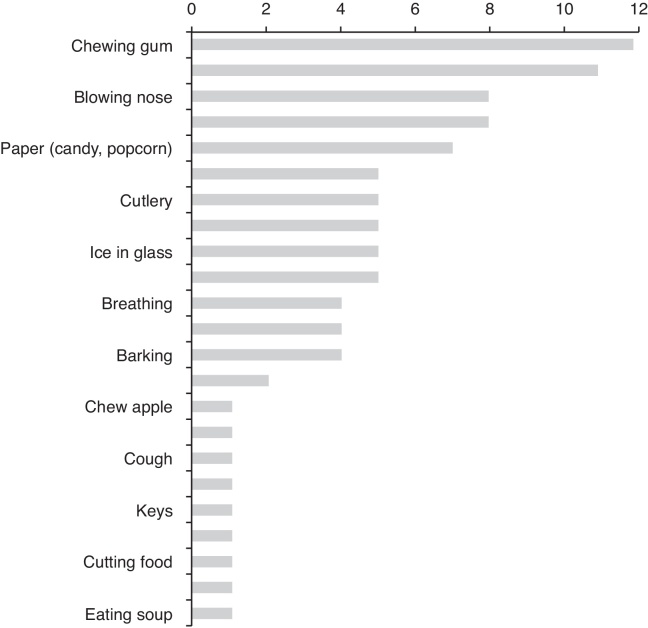
Descending order of all sounds reported by the 12 members of the family as the most important ones that trigger their misophonia.

**Figure 5:**
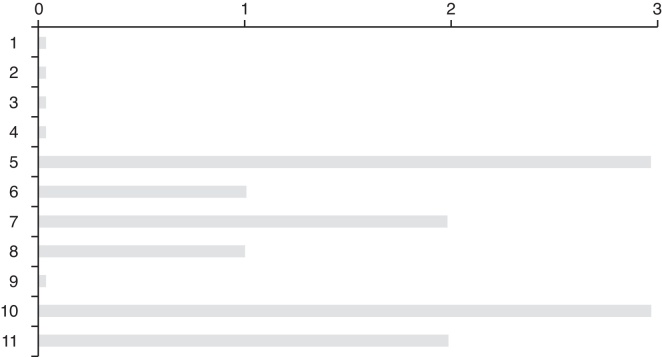
Number of specific sounds per person triggering misophonia.

Fig. 4 shows the main sounds that trigger misophonia in our sample. Fig. 5 shows the number of specific sounds per person that trigger hatred, anger or fear.

The main feelings involved in the immediate and strong emotional reactions were hatred or anger (*n* = 10; 83.3%), irritability (*n* = 3; 25%), moodiness (*n* = 1; 8.3%), discomfort (*n* = 1; 8.3%), and anxiety (*n* = 1; 8.3%).

The strategies used to face the hassle include: asking to stop the sound (*n* = 9; 75%), leaving the place (*n* = 8; 66.7%), fighting with the persons that make the sound (*n* = 7; 58.3%). Just one person tries to bear silent (*n* = 1; 8.3%).

### Impact on quality of life

When asked about whether misophonia hinder their social or professional life, 10 (83.3%) answered “sometimes”, and 2 (16.7%) answered “no”. Searching specifically whether misophonia limits their freedom to go to places where the trigger sounds are present – which seems like a limitation on quality of life – two patients answered “always”, five answered “sometimes”, and another five answered “no”.

We attempted to understand why such specific set of sounds is powerful enough to trigger such a strong emotional reaction while most sounds are not. More than one reason was applied for some participants: for 4 (33.3%) of them, the possible explanation relies on the fact that such sounds distract them in a way that blow their concentration away; 3 (25%) subjects attribute the annoyance to the fact that such sounds seem impolite, while 7 (58.3%) think that such sounds simply irritate them, without defining exactly why.

**Figure 6:**
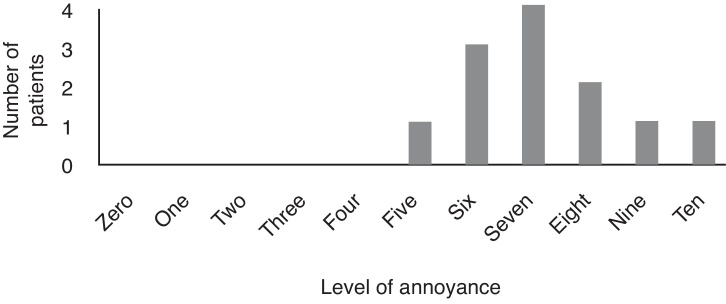
The distribution of annoyance according to the Visual Analog Scale from 0 to 10.

According to the Visual Analog Scale, the discomfort with misophonia varied from 5 to 10 (mean = 7.3; median = 7.5) (Fig. 6).

### Associated symptoms

**Figure 7:**
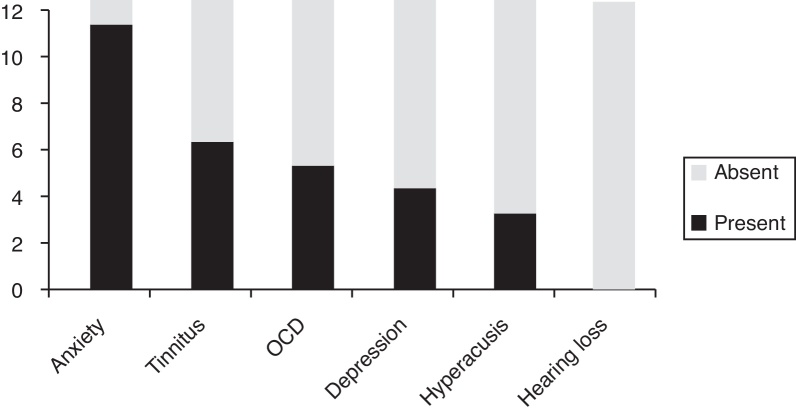
Distribution in descending order of the presence of associated symptoms in this misophonic sample (*n* = 12). OCD, Obsessive Compulsive Disorder.

The distribution of the presence/absence of associated symptoms reported by our sample is seen in Fig. 7.

### Treatment attempts

All 12 participants denied having ever sought treatment for misophonia.

## Discussion

As far as we know, there is no report about familial misophonia, although the issue has been briefly discussed previously.^3^, ^7^ The predominance of women with misophonia in this family is in agreement with other studies.^3^, ^17^ Among the 5 affected women who have had children (numbers 9, 11, 13, 20, 22), 4 had 100% of their children with misophonia (numbers 11, 13, 20, 22).

Regarding the age, both the mean (12.5 years) and the median (12 years) age of our sample correspond to the transition between childhood and adolescence. Other studies pointed to the beginning of misophonia in such time.^3^, ^18^ This was such a dominant finding in our sample that the single exception involves the female number 9 (Fig. 2).

Of special interest is the nine year-old boy (number 32), the youngest member of the family, who started his symptoms at the age of 2, according to the mother. When analyzing the younger generation IV (Fig. 2), the doubt about heredity versus environmental influence is easily evoked: in this particular case, among four people living in the same house when he was born, all presented misophonia (his mother and two sisters), except the father.

The long duration of symptoms confirms that misophonia is a chronic condition with no tendency to spontaneous improvement.

One of the most intriguing factors of misophonia is the great selectivity involving the problem, both for sounds that trigger the hassle as for the people who make the sounds. Different from patients with pure hyperacusis, usually pure misophonic subjects do not feel annoyed by loud sounds, unless both disorders coexist in the same subject. Misophonic patients have their strong and sudden emotional reaction triggered by low level, but repetitive sounds.

Based on this, the main trigger sounds were those related to mouth movements (chewing gum, chew food, brushing teeth, whistling, popping lips), nose (blowing nose, snoring, other people's breathing) or fingers (touching paper of candies/popcorn, typing, touching cutlery, clicking pen). Interesting to say, barking was commonly included in the list, challenging the definition of misophonia for those who claim that only human sounds evoke the disorder.^18^ All patients mentioned at least five common sounds that evoke strong emotional reactions.

We also investigated whether specific people caused greater discomfort than other people producing the same sounds. Half the participants indicated that their annoyance with sounds is greater when they are emitted by closely related people than by unknown. This particular aspect may be related to the type of relationship that each member of the family adopt with people around and with the freedom that participants may have to express their reactions of hatred/anger/fear in front of known/unknown persons. For the remaining six, the trouble does not depend on those who produce the sounds.

Regarding associated symptoms, it was clear that misophonia was either associated to otological/audiological symptoms (tinnitus and hyperacusis) and/or psychiatric ones (anxiety, depression, obsessive-compulsive disorder). This led us to consider that such affected members would benefit to have an extended evaluation composed of: (1) hearing exams, such as pure tone audiometry, Loudness Discomfort Levels (LDL), otoacoustic emissions and, whenever tinnitus is present, the tinnitus pitch and loudness matching; (2) psychiatric and/or psychological interventions. However, due to the long distance between the three cities where all the participants live, it was not possible to obtain such data. Due to the same reason, the presence of each associated symptom was assigned by each patient after a brief explanation, and not diagnosed by a professional.

None of the participants had ever sought treatment for misophonia. This could represent the idea that misophonia is an unknown problem, so people get used to be considered strange, weird or cranky. A similar result was previously described,^3^ in which just 2 out of 11 patients have sought treatment.

Such information could be relevant to motivate multidisciplinary effort in order to better manage this disorder, including otolaryngologists, audiologists, pediatricians, psychiatrists and psychotherapists.

If 15 consanguineous individuals report annoyance to quite similar sounds, we wonder whether misophonia would be hereditary or influenced by the environment, or both. Such numbers would favor the hypothesis of strong hereditary component. Worth to know is the spontaneous written comment on the questionnaire of one affected women: “this has already become a characteristic trace of the Andrade part of the family. It is interesting that it manifests even in the children who do not have much contact with the rest of the family”. On the other hand, most participants started to strongly react to sounds when they were children or teenagers, and this could have been learned by living with other affected members of the family.

A certain trait which is statistically linked to a family can be non-genetic, that is, transmitted from relatives to children through non-genetic pathways, but important enough to create a heredity pattern quite almost infallible. This is particularly important for the characteristics of human behavior, which can misrepresent the ideas on the etiology of psychopathology, making us believe that there are genetic causes (and therefore organic determinants, biochemical problems, histological changes) where there are only problems in the psychological domain. Cultural behavior is therefore inherited without being genetic, and misophonia could be a learned behavior in some cases.

However, it seems reasonable that one should consider that the etiology of misophonia may include the concept of a continuum of possibilities between environmental causes and heredity. If we admit that misophonia in this family is due to genetic factors, the presence of the phenotype in three generations and its transmission through affected males and females are strongly suggestive of autosomal dominant inheritance. However, the description of similar families and a systematic investigation of family history in a larger series of affected individuals would be necessary to confirm the role of genetic factors in the etiology of misophonia.

The incidence and distribution of misophonia among these family members led the researchers to suppose that the disorder may be more common than expected and likely to have autosomal dominant inheritance. Further research on familial misophonia, including twins, are welcome to confirm our findings.

## Conclusions

The distribution of 15 family members suffering from misophonia and its transmission through affected males and females strongly suggests that this disorder may have an autosomal dominant inheritance. Commonly initiated during childhood and adolescence, the strong emotional reactions involve hatred or anger to sounds and most patients avoid going or staying in places where such sounds are present.

## Conflicts of interest

The authors declare no conflicts of interest.
